# Convalescent plasma therapy in an immunocompromised patient with multiple COVID‐19 flares: a case report

**DOI:** 10.1002/rcr2.858

**Published:** 2021-11-09

**Authors:** Frini Karaolidou, Natasa‐Eleni Loutsidi, Zois Mellios, Edison Jahaj, Konstantinos Eleftheriou, Maria Pagoni, Ioannis Mpaltadakis, Athanasios‐Meletios Dimopoulos, Ioannis Kalomenidis, Apostolos G. Pappas

**Affiliations:** ^1^ Haematology – Lymphomas Department and Bone Marrow Transplant Unit Evangelismos General Hospital Athens Greece; ^2^ Covid‐19‐Dedicated Ward Evangelismos General Hospital Athens Greece; ^3^ Dermatology Department Evangelismos General Hospital Athens Greece; ^4^ 1^st^ Department of Critical Care and Pulmonary Medicine National and Kapodistrian University of Athens, School of Medicine, “Evangelismos” General Hospital Athens Greece; ^5^ Department of Clinical Therapeutics National and Kapodistrian University of Athens School of Medicine Athens Greece

**Keywords:** B‐cell acute lymphocytic leukaemia, convalescent plasma, COVID‐19, immunosuppression

## Abstract

Convalescent plasma (CP) transfusion has been utilized as a salvage therapy in immunocompromised patients with severe COVID‐19 pneumonia. We describe the case of a 45‐year‐old immunocompromised patient, who received CP, in order to control multiple COVID‐19 flares and prolonged SARS‐CoV‐2 viraemia lasting for 2 months after the initial diagnosis.

## Graphical abstract

We describe the case of a 45‐year‐old immunocompromised patient, who received convalescent plasma, in order to control multiple COVID‐19 flares and prolonged SARS‐CoV‐2 viraemia lasting for 2 months after the initial diagnosis.

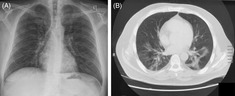

## INTRODUCTION

SARS‐CoV‐2‐infected patients with haematological malignancies are at increased risk of defective virus clearance and disease progression.^1^, ^2^ Convalescent plasma (CP) transfusion has been used as rescue therapy in immunocompromised patients during the acute phase of the disease.^3^

We report the case of a 45‐year‐old severely immunocompromised unvaccinated male patient for SARS‐CoV‐2, who received CP therapy, 2 months after the initial diagnosis, suffering from three disease flares and prolonged viraemia.

## CASE REPORT

The patient was admitted to COVID‐19‐dedicated ward of our hospital due to fever (39°C), cough, malaise, diarrhoea and positive nasopharyngeal swab real‐time quantitative reverse transcription PCR (qRT‐PCR) test for SARS‐CoV‐2. Symptoms started 2 days before his admission. That was the second episode of COVID‐19. Two months before his current admission, he had been diagnosed with SARS‐CoV‐2 infection. At that time, he presented with fever, nasal congestion, sore throat and anosmia (mild disease),^4^ lasting for 1 week. Since then, he remained asymptomatic.

He had a history of high‐risk B‐cell acute lymphocytic leukaemia (B‐ALL). Nine months before COVID‐19 diagnosis, he received allogeneic stem cell transplantation (allo‐SCT), achieving complete remission (CR) I by German Multicenter Acute Lymphoblastic Leukaemia protocol and minimal residual disease negativity following the administration of bi‐specific monoclonal antibody blinatumomab. He was transplanted from a matched unrelated donor with conditioning regimen total body irradiation/etoposide with concomitant anti‐thymocyte globulin and cyclosporine therapy, which was tapered off and ceased after 6 months. He had never developed graft‐versus‐host disease (GvHD). He received four weekly doses of rituximab, 3 months before his first admission, because of detectable Epstein–Barr virus (EBV) RNA copies in his plasma.

**FIGURE 1:**
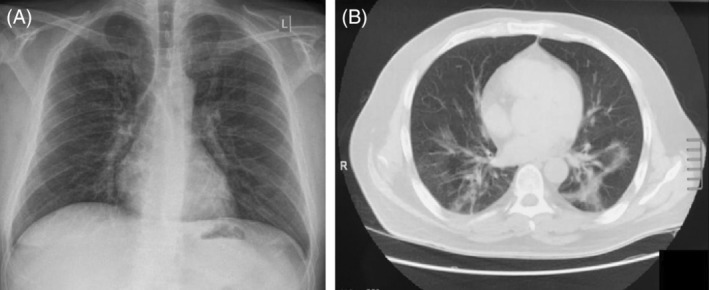
(A) Posteroanterior chest x‐ray shows bilateral ill‐defined linear opacities, mainly in the lower lung fields, L: left. (B) High‐resolution chest computed tomography shows ground‐glass opacities in the lower lung lobes, r: right

Physical examination revealed bilateral crackles at lower lung fields and fever. The rest of the vital signs were normal. Laboratory tests revealed mild elevation of C‐reactive protein (CRP, 4 mg/dl, normal value < 0.5 mg/dl) and lactate dehydrogenase (336 IU/L, normal value < 225 IU/L). Chest x‐ray (CXR) showed bilateral ill‐defined linear opacities, while chest computed tomography scan showed ground‐glass opacities in the lower lung lobes (Figure 1). qRT‐PCR for SARS‐CoV‐2 of nasopharyngeal swab and blood was positive. Although this was highly indicative of a new flare, GvHD and a variety of pathogens causing fever in an immunocompromised patient were also considered. Blood, urine, sputum and stool cultures were obtained and tested negative for bacteria, fungi, mycobacteria, *Pneumocystis jirovecii*, cytomegalovirus (CMV), EBV, adenoviruses and human herpes virus‐6. Because of diarrhoea, rectal biopsies were obtained and were not indicative for GvHD or CMV colitis. We performed bone marrow aspiration and flow cytometry (FC) confirming that the patient was in CR. The immunological recovery after allo‐SCT was estimated with FC of peripheral blood and revealed severe reduction of CD4+ T lymphocytes (45 c/μl, normal range 663–1477) and B‐cell depletion (CD19+ cells = 0/μl) with concomitant slightly elevated CD8+ T cells (997 c/μl, normal range 342–754) and normal Natural Killer cells (110 c/μl (normal range 100–350). Severe hypogammaglobulinaemia was noticed with IgG levels at 416 mg/dl (normal range 690–1618 mg/dl), IgM levels < 16.6 mg/dl (normal range 40–235 mg/dl) and IgA < 25 mg/dl (normal range 72–400 mg/dl). Anti‐SARS‐CoV‐2‐specific IgG antibodies were undetectable, suggesting that the patient was unable to mount an effective humoral response against the virus.

He was treated with prophylactic dose of enoxaparin and ceftriaxone. His symptoms and signs resolved completely by the seventh day of admission and CRP value normalized. He remained at the hospital in order to complete 21 days of clinical stability (according to the Greek National Public Health Organization Guidelines for immunocompromised host). After 11 days, fever, cough and bilateral crackles at the lower lung fields relapsed. CRP was 2.8 mg/dl. CXR did not reveal new findings. A broad microbiological survey was again negative. SpO_2_ (peripheral capillary oxygen saturation) was 97% at room air. However, viral load of SARS‐CoV‐2 in nasopharyngeal swab specimens remained high (<30 cycles in qRT‐PCR).

We assumed that the patient was under a third flare of SARS‐CoV‐2 infection, which was a result of failure to clear the virus, because of B‐cell depletion and severe impairment of T‐cell recovery. Although the disease was not progressing to severe COVID‐19,^4^ he was treated with three ABO‐compatible CP units (200 ml each given every second day). CP units were collected in the setting of the ongoing phase II, national, multicentre clinical trial evaluating plasma therapy in patients with COVID‐19 (ClinicalTrials.gov Identifier: NCT04408209) and were kindly offered to us by Prof. A. M. Dimopoulos (National and Kapodistrian University of Athens, Greece). No transfusion‐related adverse events were observed. The patient experienced a clinical improvement within 48 h from the first CP transfusion, with subsequent resolution of symptoms and signs and normalization of CRP levels. He is followed up in an outpatient weekly basis and he remains asymptomatic for more than 3 months. qRT‐PCR testing revealed a serially decreasing virus titre, reaching sustained negativity 1 month after the infusion of the first CP unit.

## DISCUSSION

We report a case of severely immunocompromised patient unable to develop effective humoral response against SARS‐CoV‐2, suffering from a protracted clinical course of COVID‐19 with multiple disease flares. For the first time, administration of CP was not used as rescue therapy; rather it was used as a means to offer passive humoral immunity in order to help the patient to eliminate the virus and avoid further COVID‐19 flares. As prolonged viral shedding in immunocompromised patients may lead to SARS‐CoV‐2 transmission, CP therapy may present an option for impeding SARS‐CoV‐2 dissemination. This is getting even more significant, if one considers the emerging data depicting that immunocompromised patients are less likely to develop an antibody response after vaccination.^5^ However, this strategy requires further validation.

## CONFLICT OF INTEREST

None declared.

## AUTHOR CONTRIBUTION

Frini Karaolidou and Apostolos G. Pappas conceived the idea. Apostolos G. Pappas overviewed the research project. Frini Karaolidou, Natasa‐Eleni Loutsidi, Zois Mellios, Edison Jahaj, Konstantinos Eleftheriou, Ioannis Kalomenidis and Apostolos G. Pappas wrote the paper. Maria Pagoni, Ioannis Mpaltadakis, Athanasios‐Meletios Dimopoulos and Ioannis Kalomenidis critically contributed to the accomplishment of this research.

## ETHICS STATEMENT

The authors declare that appropriate written informed consent was obtained for publication of this case report and accompanying images.
